# AChE Inhibition Capability of Nanogels Derived from Natural Molecules: Tannic Acid and Lysine for Alzheimer’s Disease

**DOI:** 10.3390/pharmaceutics17040502

**Published:** 2025-04-10

**Authors:** Mehtap Sahiner, Selin S. Suner, Nurettin Sahiner

**Affiliations:** 1 Department of Bioengineering, Faculty of Engineering, Canakkale Onsekiz Mart University, Terzioglu Campus, Canakkale 17100, Türkiye; sahinerm78@gmail.com; 2 Department of Chemistry, Faculty of Sciences, Canakkale Onsekiz Mart University, Terzioglu Campus, Canakkale 17100, Türkiye; sagbasselin@gmail.com; 3 Department of Bioengineering, U. A. Whitaker College of Engineering, Florida Gulf Coast University, Fort Myers, FL 33965, USA

**Keywords:** nanogel, tannic acid, lysine, antimicrobial, antioxidant, acetylcholinesterase (AChE) inhibitors

## Abstract

**Background/Objectives:** Tannic acid (TA), a known natural polyphenolic acid with many bioactivities including antioxidants, antibacterial, and antiviral, can be combined with a natural essential amino acid L-lysine (LYS) in nanogel formulations to produce p(TA-co-LYS) (p(TA-co-LYS)) nanogels. **Methods**: A 1:1 mole ratio of TA:LYS was used to prepare corresponding spherical nanogels employing formaldehyde as a linker via the Mannich reaction. **Results**: The attained p(TA-co-LYS) particles were in 283 ± 57 nm size ranges (via SEM analysis) and possessed smooth surfaces. The zeta potential measurements of p(TA-co-LYS) nanogels suspension at different solution pHs revealed the isoelectric point (IEP) of pH 4.9, suggesting that the particles are negatively charged at the physiological pH range (e.g., at 7.4). In addition to the antioxidant efficacy of nanogels confirmed by three different tests, p(TA-co-LYS) particles showed significant Fe(II) ion chelating capacity at 350 µg/mL concentrations compared to bare TA, which is 21%, whereas the LYS molecule had a chelating capacity of 100% at the same concentrations. Moreover, it was found that p(TA-co-LYS) nanogels inhibited the Acetylcholinesterase enzyme (AChE) at a concentration-dependent profile, e.g., at 333 µg/mL concentration of p(TA-co-LYS), 57.2% of the enzyme AChE activity was inhibited. Furthermore, the minimum inhibition concentrations of p(TA-co-LYS) nanogels of Gram-negative *Escherichia coli* (ATCC 8739) and Gram-positive *Staphylococcus aureus* (ATCC 6538) were determined as 12.5 mg/mL. **Conclusions**: As cytotoxicity studies of p(TA-co-LYS) nanogels on L929 fibroblast cells also ascertained that these particles can be safely used in many biomedical applications, including antioxidant materials, drug delivery devices, and enzyme inhibitors.

## 1. Introduction

Natural polyphenolic compounds such as tannic acid (TA) are plant-based active agents possessing inherently biological functions including antioxidant, antimicrobial, antiaging, antitumor, antimutagenic, anti-inflammation, anti-apoptosis, antiallergic, and antidiabetic activities [1,2]. The consumption of these micro-nutrients in the daily diet could prevent the risk of contracting some chronic diseases, especially cancer, diabetes, and neurodegenerative and cardiovascular diseases, by reducing oxidative stress, and may also have effects on enzyme mechanism and the modulation of signal transduction [2,3]. Some studies reported that polyphenols significantly affected the biochemical mechanisms of a variety of types of enzymes by the inhibition or activation of these biomolecules [4]. Thus, polyphenols could regulate blood sugar through the hormonal control of insulin and inhibition of pro-obesity enzymes [5]. Many studies show that polyphenols may inhibit digestion enzymes such as alpha-glucosidase, which is responsible for the breakdown of polysaccharide repeating units, and importantly reduce glucose absorption [6,7,8,9]. Apart from this, polyphenols could prevent or manage type 2 diabetes by triggering the B-cell growth and protection of the cells, lowering oxidative stress, and improving insulin signaling and the arrangement of gut microbiota [10]. Therefore, using polyphenolic-based formulations as antidiabetic agents could replace the traditional medical treatment agents of type 2 diabetes mellitus. Acetylcholinesterase (AChE) can be inhibited by polyphenol [11]. AChE is a serine hydrolase that stops neurotransmitter transfer at cholinergic synapses by hydrolyzing acetylcholine [12]. This enzyme, AChE, hydrolyzes acetylcholine from nerve endings, stopping nerve impulses. AChE activity increases as a result of Alzheimer’s disease, while AChE inhibitors help to alleviate Alzheimer’s symptoms [13]. TA is known to inhibit the AChE enzyme [14].

Lysine (LYS) is an essential amino acid, and it is important for protein synthesis and for various bodily functions, including bone health, immune support, hormone production, and brain development [15]. It is also particularly vital for children and adolescents, in terms of proper growth and disease prevention [16]. Additionally, LYS is crucial for several bodily functions, including the absorption of calcium, iron, and zinc, promoting collagen growth, enhancing athletic performance and muscle development, and strengthening the immune system [16]. The amine and carboxyl groups on LYS allow it to form hydrogen bonds with hydrophilic molecules, contributing to mechanical strength and adhesion [17]. Low levels of LYS have been linked to an increased risk of sarcopenia and higher mortality rates [18]. Recent studies underscore the importance of LYS succinylation in cancer and immunity [19]. This modification contributes to the aggressive traits of cancer cells, and its dysregulation is seen in multiple cancer types. Inhibitors targeting enzymes regulating LYS succinylation could provide effective cancer treatment strategies [20].

Nanogels afford innovative strategies to tackle challenges of drug transport across the blood–brain barrier (BBB) in treatments of various diseases, including Alzheimer’s disease (AD) [21]. The BBB consists of layers of vascular endothelial cells connected by tight junctions [22]. These cells are surrounded by a basement membrane and monitored by microglial cells [23]. Cohesion domains on endothelial cells resist the transport of small molecules, while transcytosis enables the movement of proteins and peptides [23]. Endothelial cells use specific transport proteins to facilitate the transport of both hydrophilic and hydrophobic molecules. Therefore, structures made up of repeating units of proteins such as amino acids have many advantages in the treatment of neurological diseases, including AD.

There is growing interest in the use of polyphenolic and amino acid-based compounds in the design of nanoformulations due to their excellent biological properties and activities, leading to their diverse utilization in biomedical applications [4]. TA and LYS have been utilized in the design of various polymeric materials, depending on their activities in the biological system [21,22,23]. In a study, a hydrogel derived from polyvinyl alcohol, poly(lysine), and tannic acid-modified Ag nanoparticles was synthesized and used as a wound dressing material. The addition of poly(lysine) and tannic acid-modified Ag nanoparticles rendered an antibacterial effect on the hydrogels and improved the wound-healing ability [24]. In a different study, tannic acid-modified poly(lysine) hydrogels were prepared for biomedical applications with highly antibacterial and antioxidant properties [25]. Garg et al. reported that a poly(lysine)-tannic acid hybrid hydrogel affords a synergistic effect, and has great bactericidal potency, leading to the inhibition of planktonic and biofilm forms of bacteria colonies [26]. In our previous studies, we reported nanoparticle preparation from polyphenol–amino acid binary mixtures, such as catechin–lysine [27], tannic acid–arginine [28], and hematoxylin–lysine [29], which were designed for biomedical purposes. Here, p(TA-co-LYS) nanogels were prepared via the Mannich condensation reaction involving TA and LYS self-assembly as two different important active agents in the treatment of ADs. TA is known as an AChE enzyme inhibitor, Fe(II) chelator, and great antioxidant material [30]. It is reacted with the essential amino acid LYS, which also grants metal-chelating abilities and neuroprotective effects [31,32]. These molecules can be combined into a polymeric nanogel formulation, e.g., p(TA-co-LYS) nanogels, that can play a vital role in the treatment of ADs. Also, infections could be triggered by the progression of neurodegenerative diseases [33], necessitating versatile materials with additional functionalities such as antibacterial properties. Nanogels prepared from antimicrobial compounds such as TA and LYS could reduce chronic infections and inflammation in the brain. The sizes and morphological and chemical characterization of the p(TA-co-LYS) nanogels were assessed by ﻿scanning electron microscopy (SEM), dynamic light scattering (DLS), Fourier transform infrared (FT-IR) spectroscopy, and zeta potential analysis. Furthermore, the antioxidant capability of the p(TA-co-LYS) nanogels was tested by comparing their potency with the standard antioxidant agents via total phenol (FC) and total flavonoid (TFC) antioxidant assays. In addition, the Fe(II) chelating ability and AChE enzyme inhibition capabilities of the prepared nanogels were tested. The antibacterial ability against Gram-negative *E. coli* and Gram-positive *S. aureus* was also determined to show the bioactivity of p(TA-co-LYS) nanogels in the biological system by comparing them with TA and LYS molecules. The biosafety of p(TA-co-LYS) nanogels was assessed by cytotoxicity analyses on L929 fibroblast cells.

## 2. Materials and Methods

### 2.1. Materials

For the p(TA-co-LYS) nanogel synthesis, tannic acid (TA, 97%), (S)-2,6-Diaminohexanoic acid (l-lysine, Ambeed, 98%, Arlington, IL, USA), and formaldehyde (FA, 37% aqueous solution) were purchased from Sigma-Aldrich, St. Louis, MO, USA. High-purity ethyl alcohol (99%, Carlo-Erba GmbH, Emmendingen, Germany) was purchased and used as received. Folin–Ciocalteau’s phenol reagent (FC, Sigma-Aldrich, St. Louis, MO, USA), sodium nitrite (Merck, extra pure), aluminum chloride (Merck, Rahway, NJ, USA, anhydrous powder sublimed from synthesis), gallic acid (GA, 97.5–102.5%, Aldrich, St. Louis, MO, USA), and rosmarinic acid (RA, 96%, Aldrich, St. Louis, MO, USA) were used for antioxidant assays. Acetylcholinesterase (AChE, Type VI-S, 200–1000 units/mg protein, Sigma, Aldrich, St. Louis, MO, USA) from the electric eel Electrophorus electricus, bovine serum albumin (BSA, Fisher Scientific, Fairlawn, NJ, USA), 5,5-dithiobis(2-nitrobenzoic acid (>98%, TCI America, Portland, OR, USA), acetylthiocholine iodide (AChI, >98%, TCI America, Portland, OR, USA), Trizma^®^hydrochloride (Sigma, Fairlawn, NJ, USA), magnesium chloride hexahydrate (Cl_2_Mg.6H_2_O, Fisher Scientific, Fairlawn, NJ, USA), and sodium chloride (NaCl, Crystalline/certified ACS, Fisher Scientific, Fairlawn, NJ, USA) were used in enzyme activity test studies.

*Escherichia coli* (*E. coli*, ATCC 8739′ KWIK-STIK, Microbiologics, Saint Cloud, MN, USA) and *Staphylococcus aureus* (*S. aureus*, ATCC 6538, KWIK-STIK, Microbiologics, Saint Cloud, MN, USA) were employed in antibacterial studies of p(TA-co-LYS). These bacteria were grown in nutrient broths (NB, Merck, Darmstadt, Germany) as a liquid medium, using nutrient agar (NA, Condolab, Madrid, Spain) as a solid medium. In the toxicity analysis of the nanogels, L929 fibroblast cells (mouse C3/An connective tissue) were supplied by a local vendor (SAP Institute, Ankara, Turkey) and the culture medium used was Dulbecco’s modified Eagle’s medium (DMEM, containing 4.5 g/L glucose, 3.7 g/L sodium pyruvate, and 0.5 g/mL L-Glutamine, PanBiontech GmbH, Aidenbach, Germany) enriched with fetal bovine serum (FBS, PanBiontech GmbH, Aidenbach, Germany) and antibiotic solution (100 IU/mL penicillin/100 μg/mL streptomycin, PanBiontech, GmbH, Aidenbach, Germany). Furthermore, trypsin/EDTA (0.25%Trypsin/0.02% EDTA, PanBiontech, GmbH, Germany), trypan blue solution (0.5%, Biological Industries, Haifa, Isreal), 3-(4,5-dimethylthiazol-2-yl)-2,5-diphenyltetrazolium bromide (MTT agent, neoFroxx GmbH, Einhausen, Germany) and dimethyl sulfoxide (DMSO, 99.9%, Carlo-Erba GmbH, Emmendingen, Germany) were used as received. Deionized water was obtained by Millipore-Direct Q UV3 (Molsheim, France) at 18.2 M.Ω.cm.

### 2.2. Synthesis of p(TA-co-LYS) Nanogel

The P(TA-co-LYS) nanogel was synthesized by the procedure described previously [27]. Briefly, 0.6 g TA was dissolved in 180 mL of EtOH: DI water (1:5, v:v) solution under a 300 rpm mixing rate. Then, 25 moles of formaldehyde per TA molecule, and 0.852 mL of 37% formaldehyde solution, were added into the mixing TA solution. After 30 min, the same mole of LYS per gallic acid unit of TA, 0.516 g in 30 mL EtOH:DI water (1:5, v:v) solution, was added to the reaction medium dropwise and reacted for 3 h under the same conditions. The suspension was centrifuged at 10,000 rpm for 10 min to precipitate the obtained p(TA-co-LYS) nanogels, and then washed with DI water two times and acetone one time to remove unreacted chemicals. The cleaned p(TA-co-LYS) nanogels were dried with a heat gun and stored in centrifuge tubes (falcon) for characterization and biological activity assessment.

### 2.3. Characterization of p(TA-co-LYS) Nanogel

A scanning electron microscope image of the p(TA-co-LYS) nanogels was acquired via SEM (SU70, Hitachi, Tokyo, Japan). Before imaging, the nanogels were coated with Pd/Au for 4 min and the image was obtained at 10 kV operating voltage.

The size measurement of p(TA-co-LYS) nanogels was performed using DLS (Brookhaven Instrument Nanobrook Omni, Holtsville, NY, USA) in a solution of 10 mM KNO_3_. The freshly prepared p(TA-co-LYS) nanogels were utilized in DLS studies or at least on the same day on which they were synthesized after washing. The hydrodynamic size analysis of p(TA-co-LYS) nanogels was carried out on freshly prepared nanogels without any drying.

The chemical structures of p(TA-co-LYS) nanogels were determined by applying Fourier Transform Infrared Radiation (FT-IR, Nicolet iS10, Thermo, Waltham, MA, USA) in a 650 to 4000 cm^−1^ spectral range at 4 cm^−1^ resolution by the ATR technique.

The thermal degradation of p(TA-co-LYS) nanogels was performed using a thermo gravimetric analyzer (TGA, SII TG/DTA 6300, Tokyo, Japan) by heating the sample from 100 to 700 °C at a heating rate of 10 °C min^−1^ under N_2_ gas at a 20 mL min^−1^ flow rate.

The determination of the zeta potential values of p(TA-co-LYS) nanogels was conducted on 1 mg/mL nanogel concentration in 1 mM KNO_3_ solution at various pHs using a Zeta Potential Analyzer (Brookhaven Instrument, BI-ZTU, Holtsville, NY, USA). In addition, the isoelectric points of p(TA-co-LYS) nanogels were determined from the zeta potential values vs. pH graphs. The pH of the solution was set by the addition of 0.2 M NaOH and 0.2 M HCl.

### 2.4. Antioxidant Properties of p(TA-co-LYS) Nanogel

Total phenol (FC) and total flavonoid (TFC) antioxidant tests were performed according to the literature [28]. In the DI water, the nanogel (P(TA-co-LYS) at 2000 µg per mL was suspended for the total phenol test. The dilution of the suspension solution was performed several times at a concentration ranging from 1000 to 125 µg per mL. A quantity of 125 µL FC solution and 20 µL sample solution were added to a 96-well plate. Then 100 µL of Na_2_CO_3_ solution in water at a concentration of 0 to 7 M was added, and the mixture was left to sit in the dark for 2 h. The absorbance value of the solution was then measured at 760 nm using a microplate reader (Thermo Scientific, Multiskan SKY, Waltham, MA, USA). The result of the antioxidant test was reported as equivalent to gallic acid (GA), since GA is the reference standard.

The total flavonoid content of the p(TA-co-LYS) nanogel was determined by testing its antioxidant properties. To this end, 50 µL of the suspended solution was added to 96 wells at a concentration range of 2000 µg per mL p(TA-co-LYS) nanogel, followed by 25 µL of 3% NaNO_2_ solution. Then, 6 percent of the AlCl_3_ solution was added to the wells. Finally, 100 mL of NaOH solution at 1 M was added to the wells. At 405 nm, the absorbance values of the solution were compared with previously determined rosmarinic acid (RA) TPC values, and the results have been expressed in mg RA equivalent in mL of total phenol.

The chelating effect of Fe(II) has been determined in the literature [27]. The TA, LYS, and P(TA-co-LYS) suspension was prepared in a stock solution of 2000 mg per mL and diluted with 1000 mg per mL DI concentrations, and diluted with 1000, 500, 250, and 125 µg per mL DI. From these solutions, 140 µL was poured onto a well-treaded plate. In each of these wells, 20 mL of 1 mM Fe(II) solution was added and absorbance values were measured at 562 nm. After adding 40 mM ferrozine to the 2 mM solution, the total volume of the solution was completed up to 200 mM and the final concentrations of the samples were 1400, 466, 233, 116.5, 58.3, and 29.1 mg per mL. The plate was measured at 562 nm using a microplate reader (Multiskan Sky, Thermofisher, Waltham, MA, USA). Then, 40 µL of 2.5 mM ferrozine solution in water was added to each of the wells. After 4 min, the plate was measured again at 562 nm using microplate readers. Water DI without a sample was used as a control. Each experiment was performed in three parts.

### 2.5. AChE Enzyme Inhibition of p(TA-co-LYS) Nanogel

AChE (EC 3.1.1.7) inhibition was calculated based on Ellman’s method with some modifications [34]. The P(TA-co-LYS) nanogels solution at a 20 mg/mL concentration was prepared in Tris -HCI buffer at pH 8 and diluted to 10,000, 5000, 2500, and 1250 µg/mL concentrations. Then, 140 µL tris-HCI buffer, 50 mM at pH 8, 20 µL of 3 mM 5,5-dithiobis (2-nitrobenzoic acid) (DTNB) in 50 mM Tris-HCI buffer containing 0.02 M MgCl_2_, 0.1 M NaCl solution, and 20 µL enzyme AChE (total enzyme 0.022 unit/mL) in 0.1% BSA solution were added to the 96-well plate. Then, 20 µL measures of the sample solution at different concentrations were added to the enzyme mixture. After 10 min, the plate was measured at 405 nm using a microplate reader. Next, 20 µL of 7.5 mM acetylthiocholine iodide as the substrate was added to each well, and the measurement of the plate was performed at 405 nm again after 20 min. The Tris-HCI buffer without the sample was used as a blank, and each experiment was performed in triplicate.

### 2.6. Antimicrobial Activity of p(TA-co-LYS) Nanogels

The antimicrobial effects of TA, LYS, and p(TA-co-LYS) nanogels were investigated by microtiter broth dilution assay against Gram-negative *Escherichia coli* (ATCC 8739) and Gram-positive *Staphylococcus aureus* (ATCC 6538). Briefly, different concentrations of TA or LYS solution or p(TA-co-LYS) nanogel suspension were prepared from 0.19 to 25 mg/mL concentrations in liquid growth medium as the nutrient broth. Then, 100 μL of this sample solution/suspension was added into the wells, and 5 μL, 1.5 × 10^8^ CFU/mL bacteria stock was added to each well. After 18–24 h incubation at 37 °C, the plate was measured at 590 nm with a microplate reader (Heales MB-530 Microplate reader, Shenzhen, China) to find the bacterial viability percentage depending on the concentration of the samples. The lowest transparent concentration was determined as the minimum inhibition concentration (MIC) value. In addition, transparent wells were inoculated on solid agar as the nutrient agar, and the lowest concentration without any bacterial growth was determined as the minimum bactericidal concentration (MBC) value. The experiment was repeated in triplicate.

### 2.7. Toxicity of p(TA-co-LYS) Nanogel

The MTT assay was performed to assess the cytotoxicity of the p(TA-co-LYS) nanogels on L929 mouse fibroblast cells at 24 h of incubation time. The fibroblasts were cultured in DMEM supplemented with 10% FBS and 1% antibiotic at 37 °C in an incubator at 5% CO_2_ atmosphere. The cell suspension was prepared at 1 × 10^5^ cell/mL in culture medium and 100 μL of the cell was added to each well of the 96-well plate. Separately, p(TA-co-LYS) nanogels were suspended in a culture medium at 1 mg/mL concentration and diluted to 500, 250, 100 and 50 μg/mL concentrations with the medium. The nanogel suspension was sterilized under a photoreactor at 420 nm (Luzchem Research Inc., Ottawa, ON, Canada) for a few minutes before contacting the cell. After 24 h incubation of the well plate to provide cell adhesion, the medium was aspirated, and 100 μL of the nanogel suspension at different concentrations was added into the well plate and incubated for 24 h and 72 h more. Then, the cells were washed with PBS once and 100 μL MTT solution at 0.5 mg/mL was added to each well. The cells were treated with MTT solution for 2 h in the dark. The MTT solution was aspirated, and 200 μL of DMSO was added into the wells and the absorbance was measured at 570 nm with a microplate reader (Heales MB-530 Microplate reader, Shenzhen, China). The experiment was run in triplicates. Statistical differences between different concentrations of p(TA-co-LYS) nanogels compared to the control group were evaluated by Ordinary one-way ANOVA Dunnett’s multiple-comparison test via GraphPad Prism 10 software. A *p*-value below 0.05 indicated a statistical difference.

## 3. Results and Discussion

**Figure 1:**
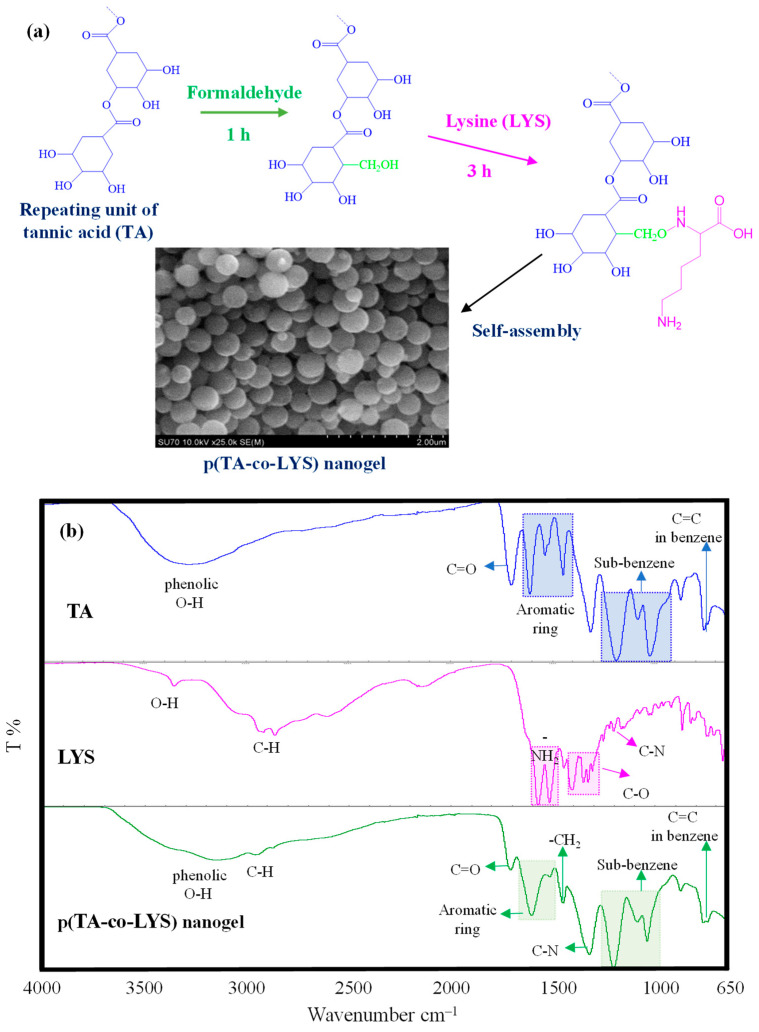
(**a**) Schematic representation of the synthesis reaction of the p(TA-co-LYS) nanogel with the Mannich reaction and SEM image of p(TA-co-LYS) nanogels. (**b**) FT-IR spectra of TA, LYS, and p(TA-co-LYS) nanogels.

Tannic acid (TA) as a naturally polyphenolic compound and lysine (LYS) as an essential amino acid were combined in p(TA-co-LYS) nanogel formulations due to the innate individual characteristics of TA and LYS that can render multiple health benefits. The presumed reaction mechanism between TA and LYS is depicted in Figure 1a. Tannic acid (TA) is a known antioxidant molecule consisting of multiple hydroxyl phenolic molecules bound to each other and the center glucose molecule. Phenolic compounds such as TA could be reacted with amino group-containing compounds, e.g., amino acids, via the Mannich reaction [35]. Earlier, we reported p(Catechin-co-Lysine) nanogels that were simply synthesized by means of the Mannich reaction [27]. In the first step, formaldehyde used as the coupling agent was bound to the C6 and C8 positions of the Catechin phenolic compound. Then, the amino group of Lysine was bound by methylene bridges, leading to a condensation reaction. As illustrated in Figure 1a, the polyphenolic compound TA is composed of gallic acid units, which resemble catechin due to the abundance of hydroxyl groups, and they could potentially react with formaldehyde in the medium in the first step. Then, the amino group of LYS can react with these phenolic aldehydes by forming methylene amine bridges through the Mannich reaction to form p(TA-co-LYS) nanogels by the spontaneous self-assembly of TA-LYS condensation products in a 20% aqueous ethanol environment. As the SEM image revealed p(TA-co-LYS), nanogels have smooth surfaces and spherical shapes, with a size range of a few hundred nanometers. The average diameters of p(TA-co-LYS) nanogels were determined as 287 ± 57 nm by observing the SEM images via the Image J software program (Image J. version 1.8.0). Also, as shown in Supplementary information as Figure S1a, the DLS measurements performed in 10 mM KNO_3_ exhibited that the hydrodynamic diameters of p(TA-co-LYS) nanogels in the swollen state were 379 ± 13 nm, with a relatively homogeneous size distribution, agreeing with the SEM images illustrated in Figure 1. The result of the energy-dispersive X-ray (EDX) analysis of p(TA-co-LYS) is given in Figure S1b. From the EDX analysis, the N atom was found to be 0.522% in p(TA-co-LYS) nanogels, indicating that LYS is incorporated into the nanogel structure.

The FT-IR spectra of TA, LYS, and p(TA-co-LYS) nanogels were compared to corroborate the chemical structures of the nanogels, as shown in Figure 1b.

Only TA has exhibited a wide peak, spanning about 3600–3000 cm^−1^, with about 3300 cm^−1^ attributed to phenolic hydroxyl group stretching vibrations. Also, the peak at 1710 cm^−1^ belongs to C=O group stretching vibrations, whereas the peak at 1603–1455 cm^−1^ is assigned to the C–C/C=C band for the aromatic phenol group, and the peaks at 1206–1040 cm^−1^ are due to the stretching vibrations of the substituted benzene rings. Finally, the peak at 763 cm^−1^ is attributed to the C=C vibration in benzene rings of TA [36,37]. The other components of the p(TA-co-LYS) nanogels, characteristic peaks of LYS, were identified at 3360, 2920, 1565–1515, 1440–1300, and 1222 cm^−1^, assigned to the -OH, C–H, -NH_2_, C–O, and C–N stretching vibrations of LYS, respectively. The peaks of phenolic hydroxyl at 3150 cm^−1^, C=O groups at 1709 cm^−1^, C=C or C–C stretching in the aromatic ring at 1603 and 1512 cm^−1^, sub-benzene at 1206–1040 cm^−1^, and C=C in benzene groups at 763 cm^−1^ of TA were also seen in the FT-IR spectra of p(TA-co-LYS) nanogels. In addition, the peak at 1324 cm^−1^ was associated with C–N stretching vibrations in the presence of amine groups derived from LYS, and the peak at 1453 cm^−1^ was related to the bending vibrations of the -CH_2_ groups in the nanogel.

**Figure 2:**
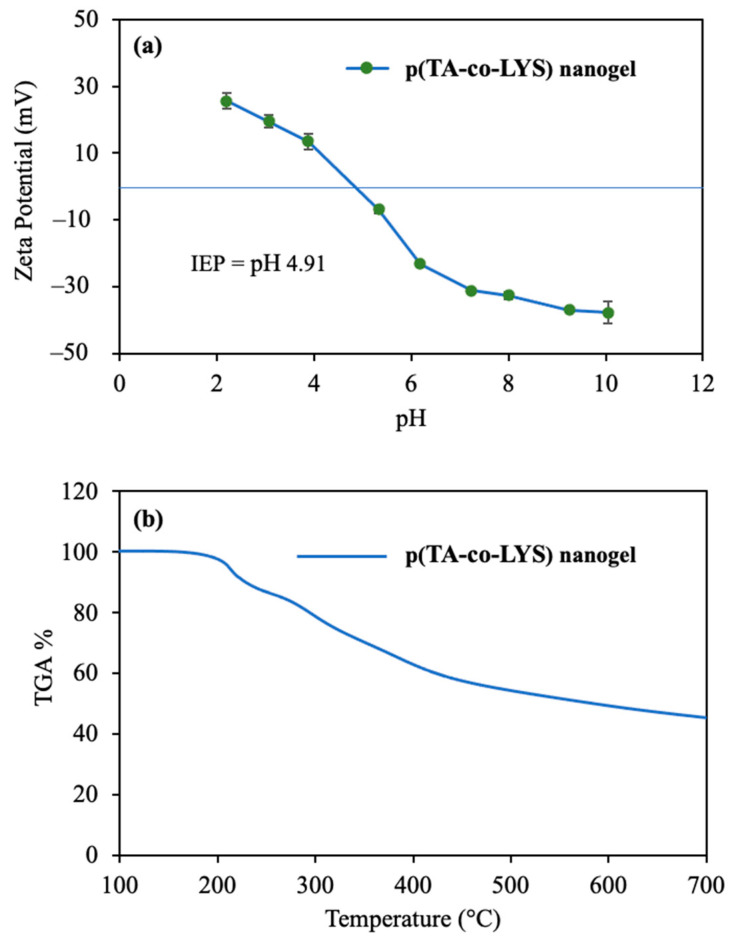
(**a**) pH versus zeta potentials of the p(TA-co-LYS) nanogel at pH 2-10 range and (**b**) thermal gravimetric analysis (TGA) or thermal degradation of the p(TA-co-LYS) nanogel.

Zeta potentials vs. solution pHs in the pH 2 to 10 range were measured for p(TA-co-LYS) nanogels, and the corresponding graph is given in Figure 2a. The zeta potential value of p(TA-co-LYS) nanogels at neutral pH (7.0) was measured as −31 ± 1 mV, suggesting that these particles are stable under physiological pH conditions. Also, the isoelectric point (IEP) of the p(TA-co-LYS) nanogels was found to be pH 4.9. This higher negative charge of p(TA-co-LYS) nanogels could afford great colloidal stability due to electrostatic repulsion. Furthermore, the p(TA-co-LYS) nanogels could be used in targeting treatments against positively charged molecules, such as some proteins or enzymes.

The thermal degradation of p(TA-co-LYS) nanogels by heating up to 750 °C was analyzed, and the corresponding thermogram is presented in Figure 2b.

As can be seen from the thermogram of the p(TA-co-LYS) nanogel, the first degradation started at about 150 °C and continued to 240 °C, with 12.1% weight loss. Then, in the second degradation temperature range, 250–430 °C, 40.7% weight loss was observed. Finally, when heating up to 700 °C, the degradation rate slightly continued, with 45.3% lost.

**Figure 3:**
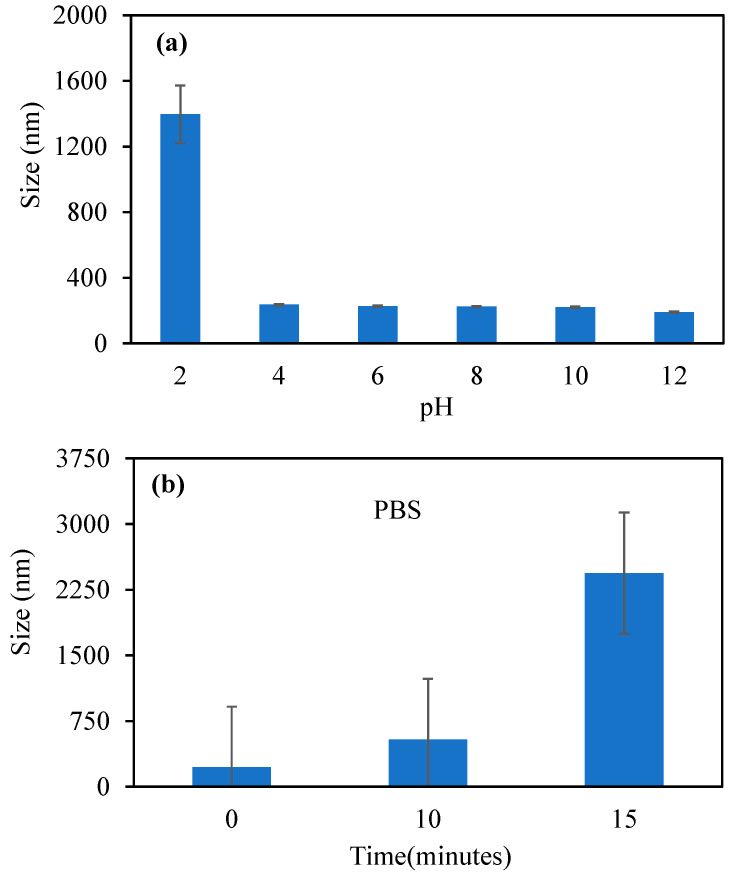
(**a**) The changes in size of p(TA-co-LYS) nanogels at different pHs via DLS measurements. (**b**) The p(TA-co-LYS) nanogel swelled in PBS solution at 7.4 pH for up to 15 min.

Figure 3a shows the changes in size of the p(TA-co-LYS) nanogels in various pH environments in the pH 2–12 range.

The size of the p(TA-co-LYS) nanogels was found to be the biggest, at 1394 nm. On the other hand, in the pH 4–12 range, the sizes of p(TA-co-LYS) nanogels were in the 235–191 nm range. The increase in solution pH caused a decrease in size at pH > 2. This is because the LYS units were deprotonated at higher pH values, which resulted in a reduction in particle sizes. In contrast, at pH 2, the LYS units in p(TA-co-LYS) nanogels are protonated, leading to bigger sizes of the nanogels. In Figure 3b, the changes in size of the p(TA-co-LYS) nanogels in PBS with time, e.g., at 0, 10, and 15 min, are given. As the sizes of the nanogels were 226 nm at time 0, and they reached 2440 nm within 15 min, this shows that p(TA-co-LYS) nanogels are highly swellable in a PBS environment. This is also in agreement with the literature, as it has been reported that polyphenol–amino acid nanogels obtained by the Mannich reaction swell in salt environments [27].

**Figure 4:**
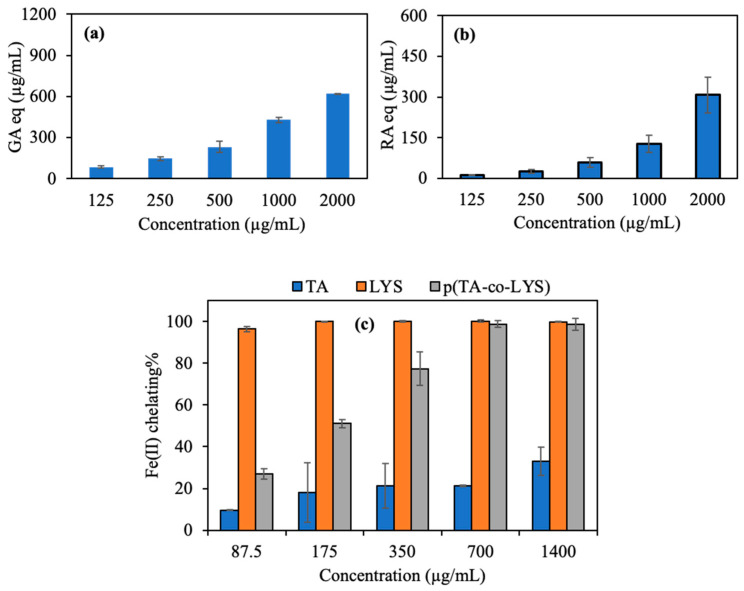
(**a**) Total phenol contents of p(TA-co-LYS) nanogels at different concentrations (125–2000 µg/mL), (**b**) total flavonoid contents of (TA-co-LYS) nanogels at different concentrations (125–2000 µg/mL), and (**c**) Fe(II) chelating activities of TA, LYS, and p(TA-co-LYS) nanogels at different concentrations (87.5–1400 µg/mL).

The antioxidant potency of the p(TA-co-LYS) nanogel, as derived from the total FC assay, is shown in Figure 4a. The total phenol content in terms of gallic acid (GA) equivalence of the p(TA-co-LYS) nanogel was determined by the concentration-dependent behavior, e.g., higher GA equivalency with higher nanogel concentration, and it reached 618.4 ± 2.5 µg/mL of GA eq at a 2000 µg/mL nanogel concentration. The overall results of the total flavonoid content (TFC) test in terms of rosmarinic acid (RA) equivalency are shown in Figure 4b. The TFC of p(TA-co-LYS) nanogels at a concentration of 2000 µg/mL reached 307.7 ± 65.7 µg/mL RA eq, suggesting that these nanogel also possess concentration-dependent antioxidant properties, e.g., the higher the concentration of the p(TA-co-LYS) nanogels, the higher the TFC values.

In Alzheimer’s and other neurological disorders, imbalances in metal ions, especially iron, significantly affect brain function. Oxidative stress and neurodegeneration can lead to excess metal accumulation [13]. Therefore, the use of chelating agents that bind excess metal ions is considered for Alzheimer’s treatment. However, their effectiveness is limited by the blood–brain barrier and toxicity. Nanostructures capable of chelating iron ions may be absorbed by apolipoprotein A-I and incubated in human plasma, aiding transport to and from the brain [38]. As demonstrated in Figure 4c, the Fe(II) chelating capacities of TA, LYS, and p(TA-co-LYS) nanogels are the highest for LYS at all investigated concentrations. As seen in Figure 4c, both TA and LYS can chelate Fe(II) ions. However, LYS amino acid chelated 96.5 ± 1.2% Fe(II) ion even at the lowest concentration (87.5 µg/mL). On the other hand, the p(TA-co-LYS) nanogel chelated 77.4 ± 8.0% Fe(II) at a 350 µg/mL concentration, showing an increased extent of Fe(II) binding with the increased amounts of p(TA-co-LYS) nanogels that were present as the LYS contents increased. Lipid and protein oxidation occurs due to free radicals. This process typically takes place as a result of a series of chain reactions, namely, initiation, propagation, and termination [39]. Certain metal ions such as Fe(II) and Cu(II)) facilitate electron transfer, leading to an augmented formation of free radicals and catalyzing lipid and protein oxidation. Lysine and p(TA-co-LYS), as reported here, may prevent lipid and protein oxidation via their Fe(II) ion chelating ability and innately antioxidant characteristics.

**Figure 5:**
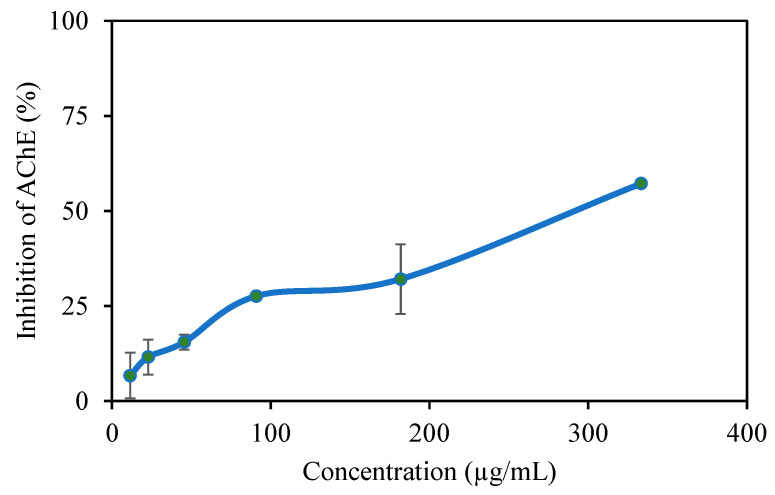
The change of in AChE inhibition (%) with p(TA-co-LYS) nanogels concentration.

Alzheimer’s disease (AD) is a progressive neurodegenerative disorder of the central nervous system that causes dementia [11]. TA has been reported as an AChE enzyme inhibitor [40,41]. Figure 5 shows the AChE enzyme inhibition graph of the p(TA-co-LYS) nanogel at varying concentrations. P (TA-co-LYS) nanogels inhibit the AChE enzyme to an increasing extent with an increase in the concentrations of the nanogels. For example, at a 333 μg/mL concentration, p(TA-co-LYS), the nanogels inhibit 57.2 ± 1.2% of the enzyme. The IC50 was determined at 292 µg per mL.

**Figure 6:**
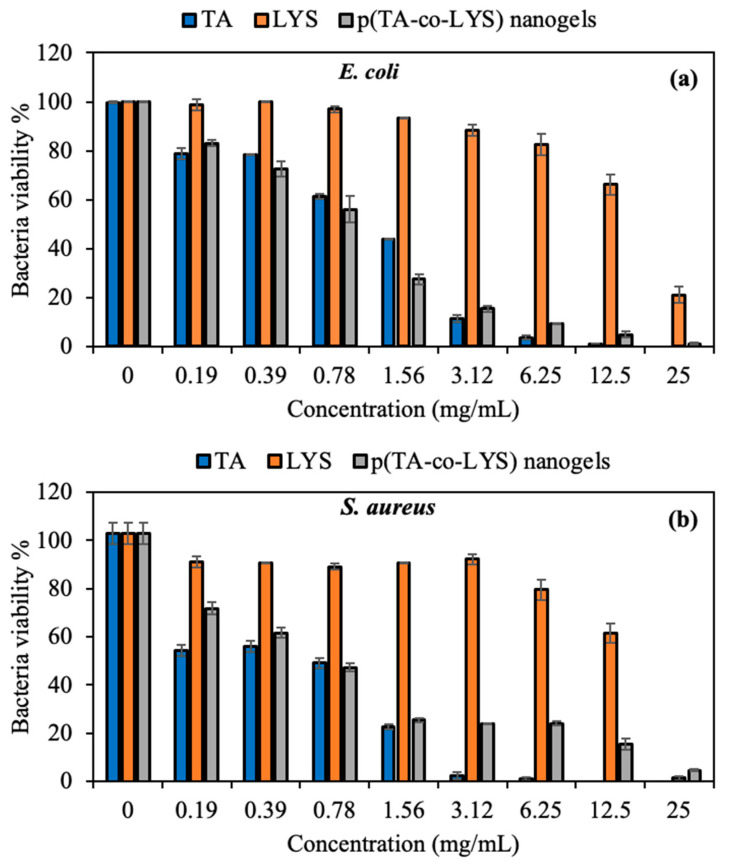
Bacteria viability percentage of (**a**) *Escherichia coli* and (**b**) *Staphylococcus aureus* treated with TA, LYS, and p(TA-co-LYS) nanogels at various concentrations from 0.19 to 25 mg/mL for 24 h.

The antibacterial activities of TA, LYS, and p(TA-co-LYS) nanogels were investigated against Gram-negative *Escherichia coli* (*E. coli*, ATCC 8739) and Gram-positive *Staphylococcus aureus* (*S. aureus*, ATCC 6538) species by microtiter dilution tests. The viability percentages of the bacteria in the presence of different concentrations of TA, LYS, and p(TA-co-LYS) nanogels are demonstrated in Figure 6.

It is well-known that natural polyphenols, such as TA, exhibit antimicrobial activity against a wide range of bacteria, fungi, or viral pathogens [42]. As indicated in Figure 6a,b, TA significantly inhibited the bacteria colony at a 3.12 mg/mL concentration, with only 11.5 ± 1.5% viability of *E. coli* and 2.2 ± 1.5% viability of *S. aureus*. The MIC and MBC values of the TA, LYS, and p(TA-co-LYS) nanogels against *E. coli* and *S. aureus* are also given in Table 1.

As seen in Table 1, the MIC values of TA were found to be 6.25 and 1.56 mg/mL against *E. coli* and *S. aureus*, respectively. Furthermore, the MBC, which is the total bacterial inhibition concentration, of TA was determined as 25 and 12.5 mg/mL against *E. coli* and *S. aureus*, respectively. These results indicate that TA had a greater antibacterial effect on Gram-positive than Gram-negative species. Interestingly, a very high concentration of LYS, e.g., 25 mg/mL, showed significant antibacterial effects on *E. coli*, with 21.0 ± 3.3% viability, and it showed 1.4 ± 0.9% viability for *S. aureus*. As listed in Table 1, LYS did not exhibit any MIC value against *E. coli* up to a 25 mg/mL concentration, but a high MIC value was found for LYS against *S. aureus* at 25 mg/mL. The bacteria inhibition effects of the prepared p(TA-co-LYS) nanogels were similar to those of TA, as seen in Figure 6. According to the results, p(TA-co-LYS) nanogels inhibited more than 75% of bacterial colony growth for both Gram-negative and Gram-positive bacteria at a 3.12 mg/mL concentration. The MIC value of the p(TA-co-LYS) nanogels was found to be 12.5 mg/mL, with 4.8 ± 1.3% viability against *E. coli* and 15.5 ± 2.2% against *S. aureus*. Polyphenolic derivates such as TA can readily interact with the cell membrane component of various bacteria, as TA possesses some lipophilic characteristics due to its structures; it is thus considered amphipathic and damages the cell wall. Therefore, TA derivates cause the leakage of essential cellular components and inhibit the bacterial colony. In addition, TA has a high binding affinity for different proteins and enzymes of the bacteria, and has the ability to diminish their activity because of its phenolic hydroxyl groups [37]. The antibacterial mechanisms of the p(TA-co-LYS) nanogels could be attributed to the bacterial inhibition ability of TA inside the nanogel polymeric network.

These results indicate that p(TA-co-LYS) nanogels have significant potential use as an antibacterial agent with reasonable antibacterial activity against both types of bacteria.

TA is a well-known active agent with inherently antioxidant and antimicrobial properties, but the toxicity of healthy cells limits its use in a wide range of biomedical applications. As previously reported by our group, TA has some toxicity even at 10 μg/mL concentration, with more than 75% cell viability [37]. TA shows level-1 toxicity at 10 μg/mL, but a high concentration of TA up to 150 μg/mL displayed level-2 toxicity, with approximately 50% cell viability [37], according to the ISO-10993-5 standard (ISO 10993.12-2005) [43], and the results show that TA has a dose-dependent cytotoxic effect on L929 fibroblasts. Also, LYS is a biomolecule, an essential amino acid that is considered biocompatible and non-toxic to cells. L929 fibroblast cells are generally used in toxicity analyses of biomaterials that have potential use in pharmaceuticals, wound dressing, or medical devices. The cytotoxicity of p(TA-co-LYS) nanogels was also evaluated on L929 fibroblast cells, obtained from the subcutaneous tissues of mice.

**Figure 7:**
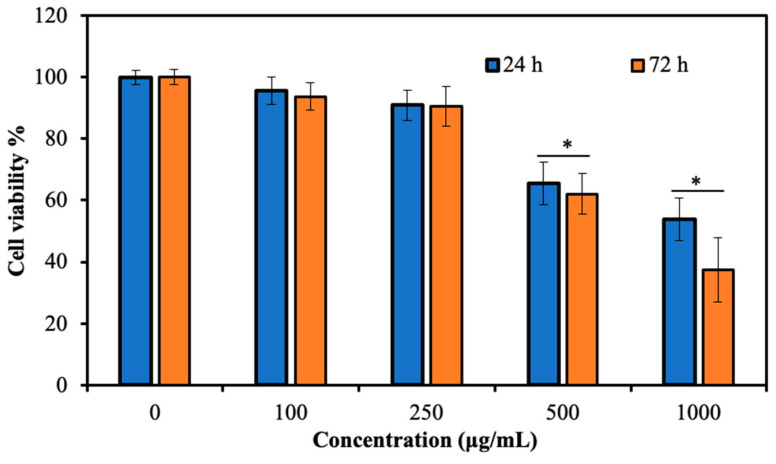
Cell viability percentages of L929 fibroblast cells interacting with different concentrations of p(TA-co-LYS) particles for 24 h and 72 h incubation times. Statistical analysis results expressed as * *p* < 0.05.

As seen in Figure 7, p(TA-co-LYS) nanogels show high biocompatibility up to 250 μg/mL concentration, with 91 ± 5% cell viability at 24 h incubation time for fibroblast cells. Even at 72 h incubation time, the cytotoxicity of p(TA-co-LYS) nanogels did not significantly change at the 250 μg/mL concentration, with a 90 ± 6% cell viability for fibroblasts. However, at high concentrations of p(TA-co-LYS) nanogels, e.g., >500 μg/mL, the cells were destroyed, with level-2 toxicity, at a 24 h incubation time, and level-3 toxicity at a 2 h incubation time. These results show that p(TA-co-LYS) nanogels could be safely implemented in biomedical areas up to 250 μg/mL concentrations.

## 4. Conclusions

p(TA-co-LYS) nanogels were successfully synthesized through the Mannich reaction between TA and LYS in the presence of formaldehyde. The prepared p(TA-co-LYS) particles were about 250 nm in size, and were spherical. It was found that p(TA-co-LYS) nanogels can readily disperse in biological fluids due to their highly negative surface charge, which is ~31 mV in the physiological pH range. The p(TA-co-LYS) nanogels have also shown high potential use as therapeutic agents to be used in the treatment of neurodegenerative diseases such as Alzheimer’s disease, due to their antioxidant and Fe(II) ion chelating capacity, as well as their AChE enzyme-inhibiting abilities. Similar to TA, here, p(TA-co-LYS) nanogels significantly inhibited the bacterial growth of Gram-negative and Gram-positive bacteria, even at 1.56 mg/mL concentration, demonstrating excellent antibacterial activity. Furthermore, no statistical difference was found in the cytotoxicity of p(TA-co-LYS) nanogels up to a 250 μg/mL concentration compared with the control group, and p(TA-co-LYS) nanogels were found to be safer than TA in the presence of fibroblast cells even at higher concentrations. Therefore, the nanogels of p(TA-co-LYS) derived from a natural phenolic compound (TA) and amino acid (LYS) provide new opportunities and possibilities for use in the biomedical and pharmaceutical fields.

## Figures and Tables

**Table 1:** Minimum inhibition concentration (MIC) and minimum bactericidal concentration (MBC) values of TA, LYS, and p(TA-co-LYS) nanogels against *Escherichia coli* and *Staphylococcus aureus*.

Sample	*E. coli*		*S. aureus*	
	MIC (mg/mL)	MBC (mg/mL)	MIC (mg/mL)	MBC (mg/mL)
**TA**	6.25	25.00	1.56	12.50
**LYS**	N.D.	N.D.	25.00	N.D.
**p(TA-co-LYS) nanogels**	12.50	N.D.	12.50	N.D.

## Data Availability

All data generated in this research are contained within this article.

## Supplementary Materials

The following supporting information can be downloaded at: https://www.mdpi.com/article/10.3390/pharmaceutics17040502/s1, Figure S1: (a) P(TA-co-LYS) nanogel size distribution by DLS measurement, (b) Energy-dispersive X-ray spectroscopy analysis of p(TA-co-LYS) nanogels.
