# Supplementary material for: AChE Inhibition Capability of Nanogels Derived from Natural Molecules: Tannic Acid and Lysine for Alzheimer’s Disease
Source: Pharmaceutics. 2025 Apr 10;17(4):502. doi: 10.3390/pharmaceutics17040502 (PMC12030174; doi:10.3390/pharmaceutics17040502)
Supplement: Supplementary file 1 [file pharmaceutics-17-00502-s001.zip › pharmaceutics-3529857-supplementary.pdf]

AChE inhibition capability of nanogels derived from natural molecules: tannic acid and lysine for Alzheimer's Disease

Mehtap Sahiner [1], Selin S. Suner [2] and Nurettin Sahiner [2,3,*]

[1] Department of Bioengineering, Faculty of Engineering, Canakkale Onsekiz Mart University, Terzioglu Campus, Canakkale, 17199, TURKEY; sahinerm78@gmail.com
[2] Department of Chemistry, Faculty of Sciences, Canakkale Onsekiz Mart University, Terzioglu Campus, 17100, Canakkale, TURKEY; sagbasselin@gmail.com ; sahiner71@gmail.com
[3] Department of Bioengineering, U. A. Whitaker College of Engineering, Florida Gulf Coast University, Fort Myers, 33965, FL, USA. (sahiner71@gmail.com; nsahiner@fgcu.edu)

* Correspondence: sahiner71@gmail.com; nsahiner@fgcu.edu

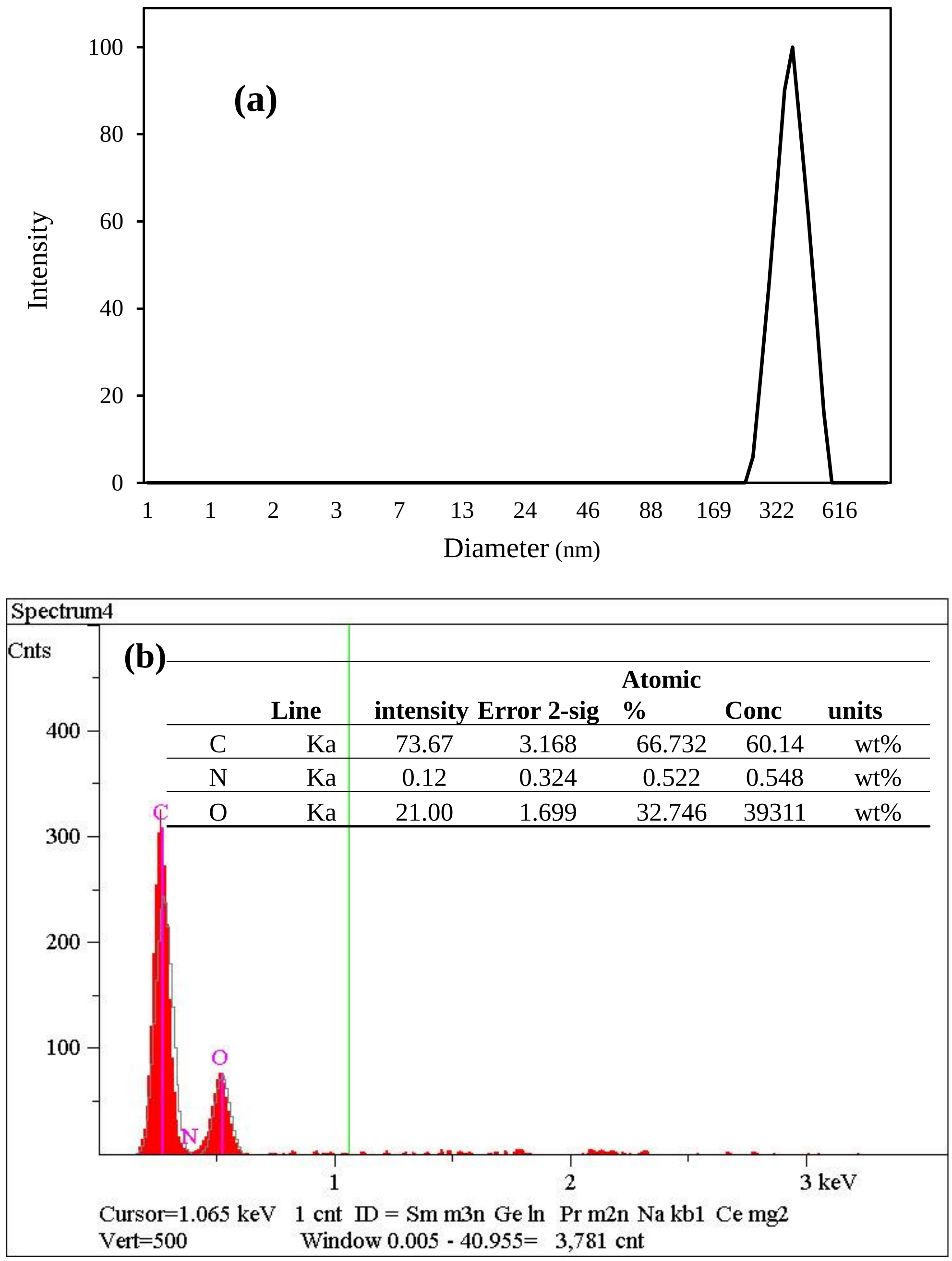

Supplementary Figure S1. (a) P(TA-co-LYS) nanogel size distribution by DLS measurement, **(b)** Energy-dispersive X-ray spectroscopy analysis of p(TA-co-LYS) nanogels.
